# Supplementary material for: A Customizable and Low-Cost Ultraviolet Exposure System for Photolithography
Source: Micromachines (Basel). 2022 Dec 1;13(12):2129. doi: 10.3390/mi13122129 (PMC9788398; doi:10.3390/mi13122129)
Supplement: Supplementary file 1 [file micromachines-13-02129-s001.zip › micromachines-2048584-supplementary.pdf]

micromachines

MDPI

Article

A Customizable and Low-Cost Ultraviolet Exposure System for Photolithography

David Eun Reynolds [1], Olivia Lewallen [2], George Galanis [1] and Jina Ko [1,3,*]

[1] Department of Bioengineering, University of Pennsylvania, Philadelphia, PA 19104, USA
[2] Department of Biomedical Engineering, Boston University, Boston, MA 02215, USA
[3] Department of Pathology and Laboratory Medicine, University of Pennsylvania, Philadelphia, PA 19104, USA
* Correspondence: jina.ko@pennmedicine.upenn.edu

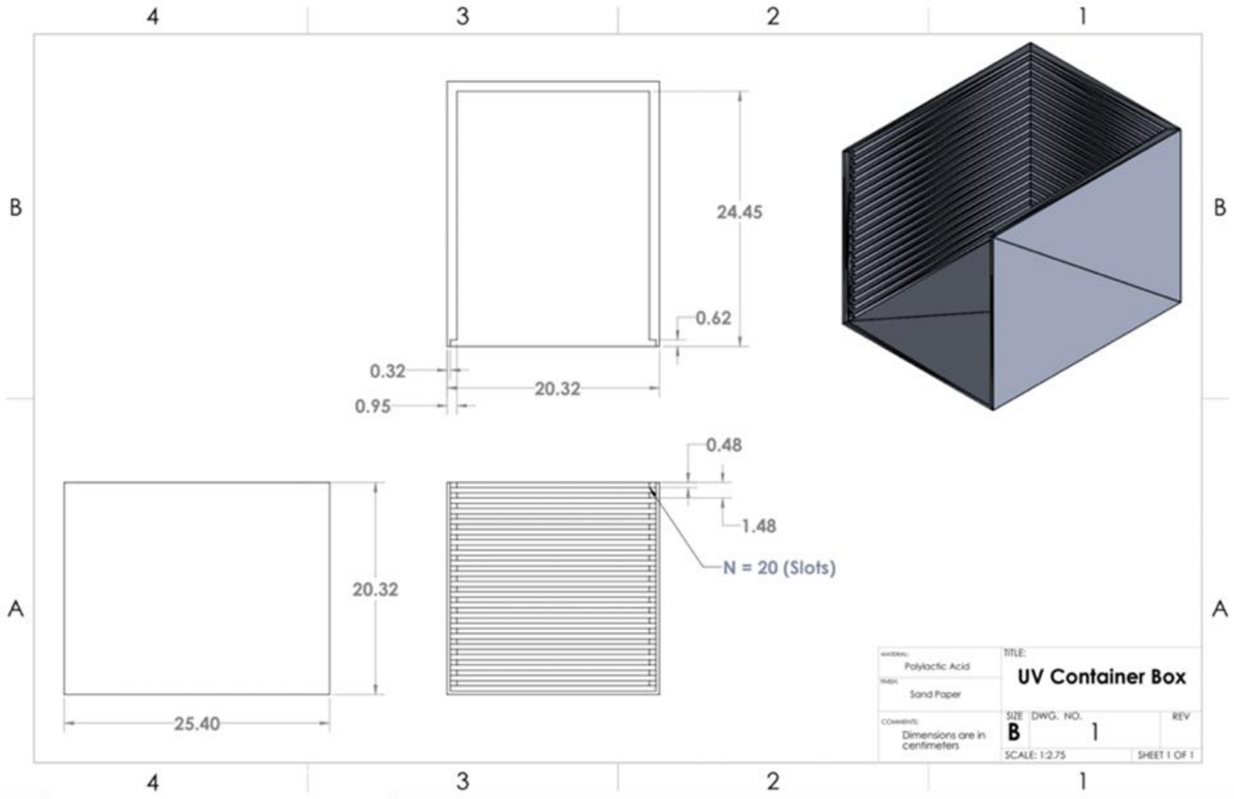

Figure S1. UV containment unit CAD drawing.

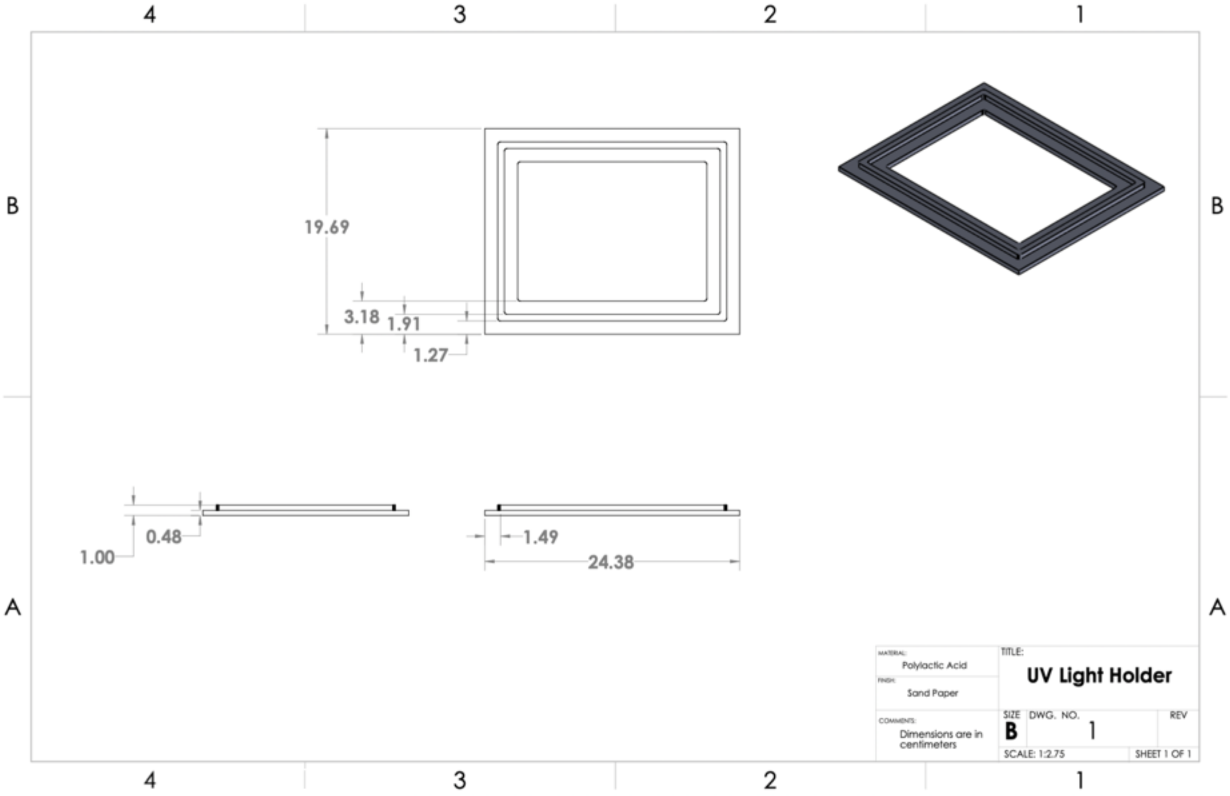

Figure S2. UV light holder CAD drawing.

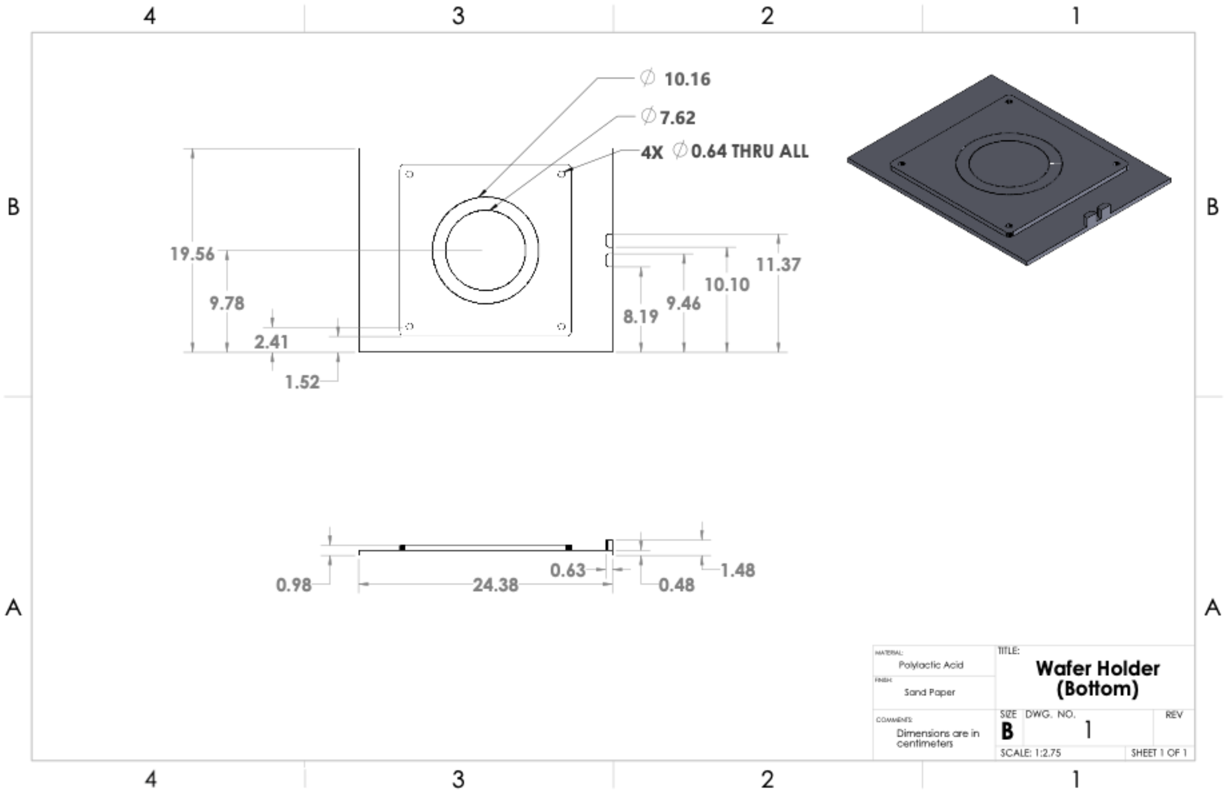

Figure S3. Bottom wafer houlder CAD drawing.

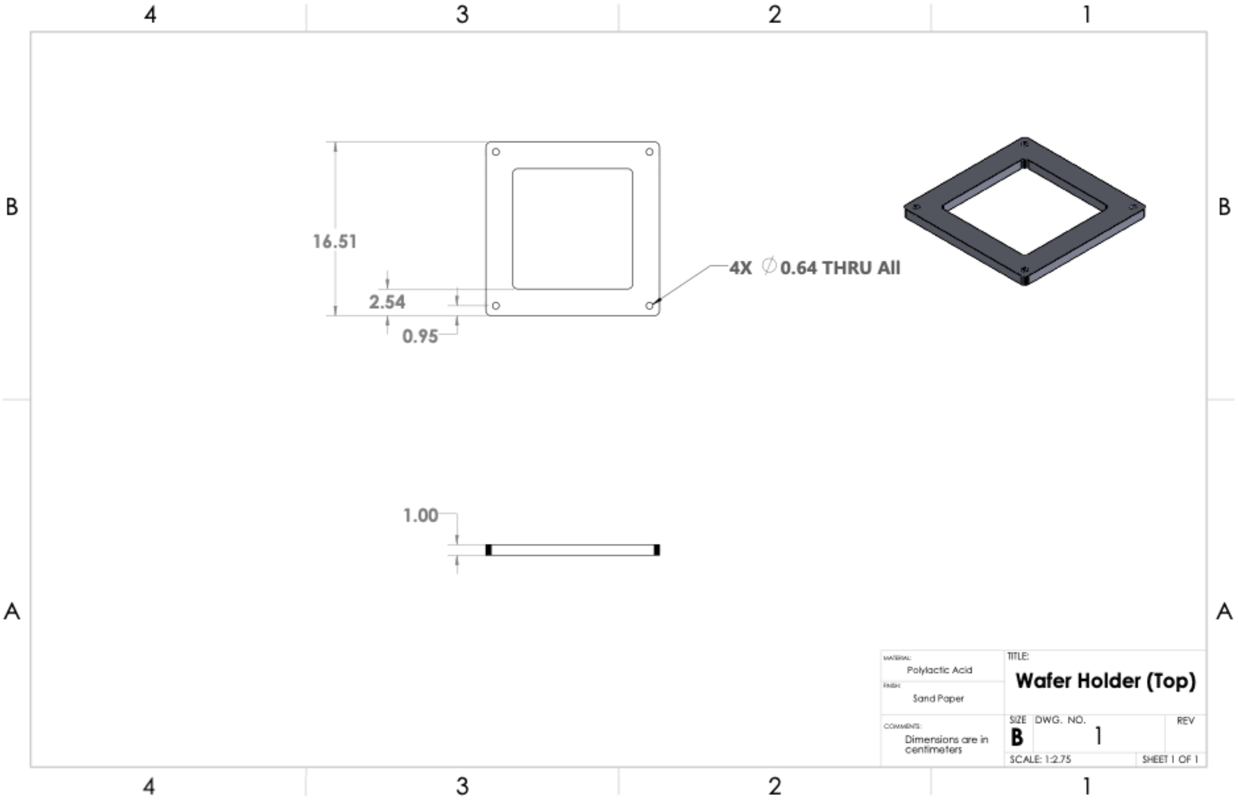

Figure S4. Top wafer holder CAD drawing.

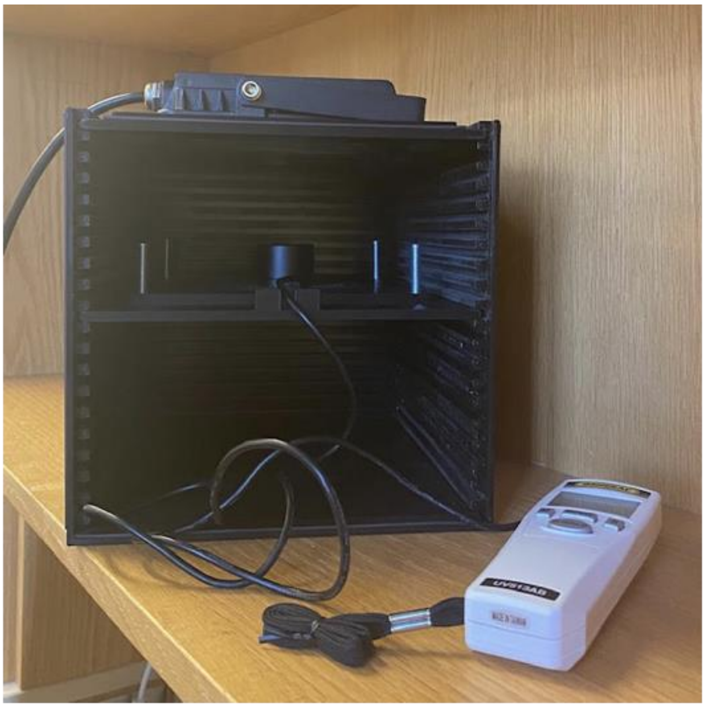

Figure S5. Calculating curing times and verifying the quality of the UV light exposure with the UV intensity meter in the UV containment unit.

UV Curing Guide				
**Thickness of Polymer (microns)**	**Intensity Required (mJ/cm^2)**			
4-10	100-200			
8-15	125-200			
20-50	150-250			
30-80	150-250			
40-100	150-250			
**Distance from UV Light**	**Curing Time for 100 mJ/cm^2**	**Curing Time for 150 mJ/cm^2**	**Curing Time for 200 mJ/cm^2**	**Curing Time for 250 mJ/cm^2**
1 cm	0 minutes 5 seconds	0 minutes 8 seconds	0 minutes 11 seconds	0 minutes 14 seconds
2 cm	0 minutes 6 seconds	0 minutes 9 seconds	0 minutes 12 seconds	0 minutes 15 seconds
3 cm	0 minutes 8 seconds	0 minutes 13 seconds	0 minutes 17 seconds	0 minutes 22 seconds
4 cm	0 minutes 10 seconds	0 minutes 16 seconds	0 minutes 22 seconds	0 minutes 28 seconds
5 cm	0 minutes 13 seconds	0 minutes 21 seconds	0 minutes 29 seconds	0 minutes 39 seconds
6 cm	0 minutes 15 seconds	0 minutes 24 seconds	0 minutes 34 seconds	0 minutes 45 seconds
7 cm	0 minutes 21 seconds	0 minutes 33 seconds	0 minutes 48 seconds	1 minute 4 seconds
8 cm	0 minutes 24 seconds	0 minutes 39 seconds	0 minutes 56 seconds	1 minute 15 seconds
9 cm	0 minutes 30 seconds	0 minutes 50 seconds	1 minute 12 seconds	1 minute 35 seconds
10 cm	0 minutes 35 seconds	1 minute 0 seconds	1 minute 27 seconds	1 minutes 56 seconds

Figure S6. User-friendly chart for identifying the proper curing time according to the user's desired outcome.

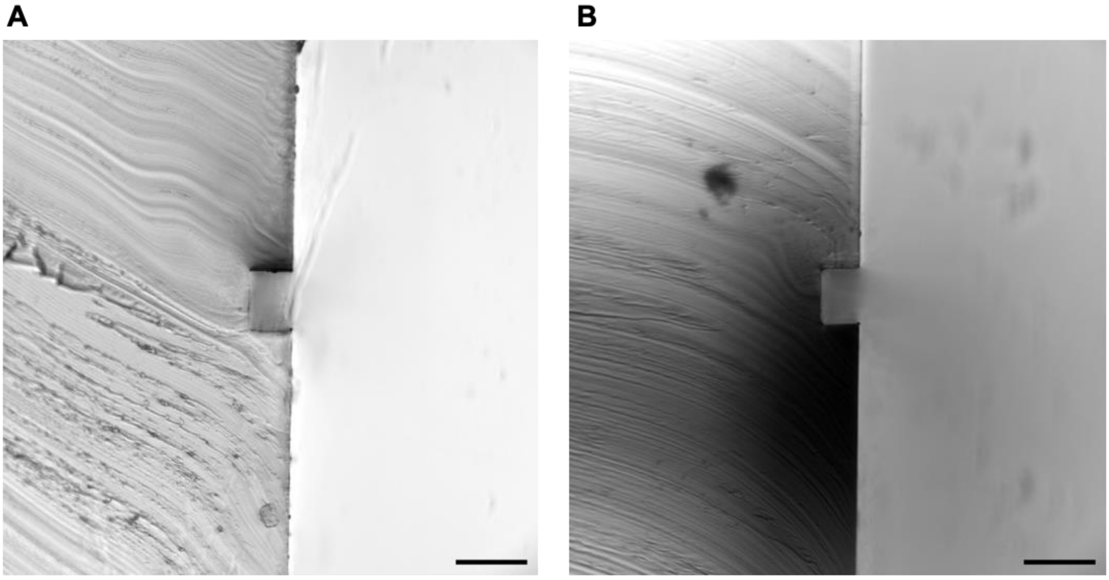

Figure S7. Cross-sections of (**A**) ABM 3000HR Mask Aligner and (**B**) DUV's T-Junction microfluidic devices. Edge quality of the produced microstructures are highlighted by the cross-section images. (Scale bar = 100 μm).

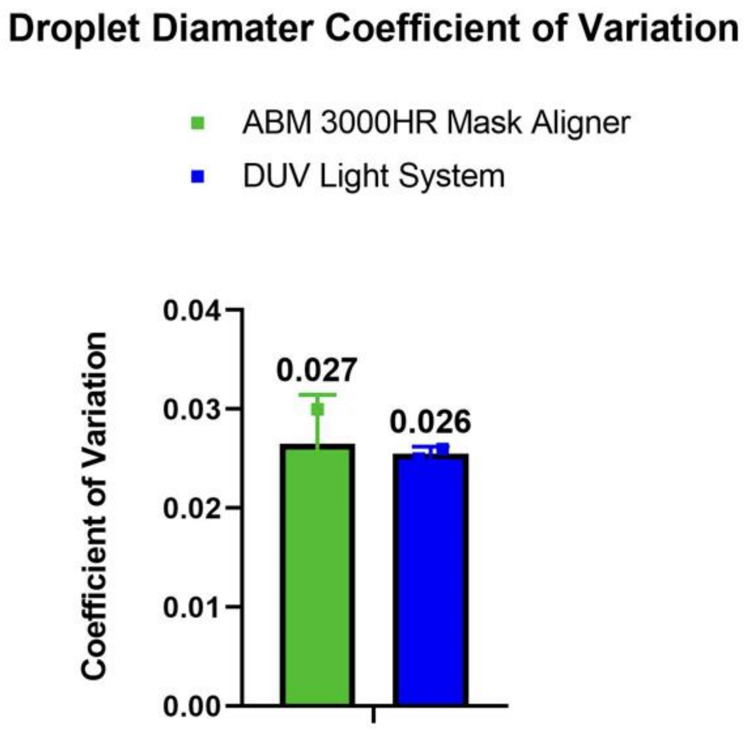

Figure S8. The droplet diameter coefficient of variation between the DUV Light System and ABM 3000HR Mask Aligner.
